# Risk of higher dose methotrexate for renal impairment in patients with rheumatoid arthritis

**DOI:** 10.1038/s41598-020-75655-9

**Published:** 2020-10-30

**Authors:** Keigo Hayashi, Ken-Ei Sada, Yosuke Asano, Sumie Hiramatsu Asano, Yuriko Yamamura, Keiji Ohashi, Michiko Morishita, Haruki Watanabe, Mariko Narazaki, Yoshinori Matsumoto, Jun Wada

**Affiliations:** Department of Nephrology, Rheumatology, Endocrinology and Metabolism, Okayama University Graduate School of Medicine, Dentistry and Pharmaceutical Sciences, 2-5-1 Shikata-cho, Kitaku, Okayama City, 700-8558 Japan

**Keywords:** Nephrology, Rheumatology

## Abstract

Renal impairment is a major concern in patients taking high-dose methotrexate (MTX) for malignancy, but it has not been fully explored in rheumatoid arthritis (RA) patients taking low-dose MTX. This study aimed to elucidate the dose-dependent effects of MTX on the renal function of patients with RA. We retrospectively reviewed 502 consecutive RA patients who were prescribed MTX for ≥ 1 year at Okayama University Hospital between 2006 and 2018. The primary outcome was the change in estimated glomerular filtration rate (eGFR) over 1 year. The association between MTX dosage (< 8, 8–12, and ≥ 12 mg/week) and the change in eGFR was evaluated using multiple linear regression analysis with adjustment for possible confounding factors including age, sex, disease duration, body weight, comorbidity, baseline eGFR, concomitant treatment, and disease activity. Mean patient age was 63 years; 394 (78%) were female. Median disease duration was 77 months, while mean MTX dosage was 8.6 mg/week. The last 1-year change of eGFR (mean ± SD) in patients treated with MTX < 8 (n = 186), 8–12 (n = 219), ≥ 12 mg/week (n = 97) decreased by 0.2 ± 7.3, 0.6 ± 8.6, and 4.5 ± 7.9 mL/min/1.73 m^2^/year, respectively (p < 0.0001). After adjustment for the confounding factors, MTX ≥ 12 mg/week was still correlated with a decrease in 1-year eGFR (beta-coefficient: − 2.5; 95% confidence interval, − 4.3 to − 0.6; p = 0.0089) in contrast to MTX 8–12 mg/week. Careful monitoring of renal function is required in patients with MTX ≥ 12 mg/week over the course of RA treatment regardless of disease duration.

## Introduction

The last decade has been seen the drastic paradigm shifts in the treatment of rheumatoid arthritis (RA). Few efficient conventional synthetic disease-modifying antirheumatic drugs (csDMARDs) have been developed, of which methotrexate (MTX) became available first, followed by biological agents. Among those treatment options, MTX is the promised anchor drug that should be firstly considered for use in patients diagnosed with RA^1^. Although it is recommended that MTX be rapidly escalated to the maximum tolerated dosage (25–30 mg/week), the optimal dosage might be lower for Asian patients^1–3^.

Chronic kidney disease (CKD) occurs more frequently in patients with RA than in the general population^4,5^. Previous reports showed that with treatment options, such as bucillamine and nonsteroidal anti-inflammatory drugs (NSAIDs)^4,6^, secondary amyloidosis^6^ and chronic inflammation^7^ were risk factors for renal impairment in RA patients, while the use of biological DMARDs was associated with a lower risk of renal impairment^8^. Although renal impairment is a major concern in patients taking high-dose MTX for the treatment of malignancy, it has not been fully examined in RA patients treated with low-dose MTX. A case series of patients with RA suggested that renal impairment was caused by MTX^9^, whereas another showed that MTX and its dosage were not associated with the detection of abnormal estimated glomerular filtration rate (eGFR) (< 90 mL/min/1.73 m^2^)^10^.

This study aimed to elucidate the dose-dependent effects of MTX on renal function by evaluating the association between MTX dosage and the 1-year change of eGFR in RA patients.

## Patients and methods

### Study design and patient selection

We retrospectively reviewed 502 consecutive patients with RA who were prescribed oral MTX for ≥ 1 year at Okayama University Hospital between April 2006 and March 2018. All patients fulfilled American College of Rheumatology/European League Against Rheumatism 2010 Classification Criteria for RA^11^. Exclusion criteria were: (1) < 20 years of age, (2) nephrotic syndrome, (3) unilateral kidney, and (4) rheumatic disorders other than secondary Sjögren’s syndrome.

### Data collection

Clinical data during the most-recent 1-year exposure to MTX between April 2006 and March 2018 was collected through electronic medical records. The following information was collected at start of the observational period as baseline data: age, sex, disease duration, body weight, body mass index (BMI), prednisone use and dose, NSAID use, use of csDMARDs, use of biological DMARDs (bDMARDs), use of targeted synthetic DMARDs (tsDMARDs), hypertension (receiving treatment and/or blood pressure above 140/90 mmHg), diabetes (receiving treatment and/or Hemoglobin A1c [HbA1c] above 6.5%), secondary Sjögren’s syndrome, white blood cell (WBC) count, hemoglobin (Hb), platelet count, serum creatinine (s-Cr) levels, proteinuria (> 0.5 g/gCr and/or ≥ 2 + on dipstick urinalysis), erythrocyte sedimentation rate (ESR), C-reactive protein (CRP) level, 28-joint Disease Activity Score (DAS28)-CRP, Simplified Disease Activity Index (SDAI), Clinical Disease Activity Index (CDAI), and Health Assessment Questionnaire (HAQ) results. MTX dosage was calculated as the mean dosage (mg/week) during the 1-year observational period.

### Outcome

The primary outcome measure was the change in eGFR during the previous 1 year for each patient. The eGFR was calculated by the equation defined by the Japanese Society of Nephrology: eGFR (mL/min/1.73 m^2^) = 194 × (serum creatinine [mg/dl])^−1.094^ × (age)^−0.287^ × 0.739 (if female)^12^.

### Statistical analysis

Clinical characteristics are presented as mean ± standard deviation (SD) or median (interquartile range; IQR) for continuous variables and patient number (%) for categorical variables. One-way analysis of variance (one-way ANOVA) was used for continuous variables, and Fisher’s exact tests were used for categorical variables to compare outcomes and patient characteristics among patients with different MTX dosages: < 8, 8–12, and ≥ 12 mg/week. The cutoff for the dosage of MTX was determined by the reference of Japan College of Rheumatology guideline for the use of methotrexate in patients with rheumatoid arthritis; 8 mg, at which is recommended as the initial dose, 12 mg, to which is recommended in patients showing inadequate response to the initial dose of MTX, and further dose-escalation of MTX to 16 mg/week (approved maximum dose in Japan) was optional according to the risk–benefit in each patient^13^.

To adjust for confounding factors using multiple linear regression analysis, possible confounders were selected according to the univariate analysis results and the findings of previous reports on risk factors on renal impairment. As for MTX dose groups with significantly higher risk of renal impairment in the regression analysis, the change of eGFR was followed for the extended 5-year period in patients whose laboratory data could be obtained, in order to observe the accumulative effect. Missing data for disease duration, body weight, BMI, ESR, and DAS28-ESR/CRP, and HAQ findings were imputed by multivariate normal imputation using the least squares method.

Statistical testing was two-sided, and values of *p* < 0.05 were considered statistically significant. For the comparison of three categories, statistical significance was determined by *p* < 0.05/3 using Bonferroni correction to avoid multiplicity. All statistical analyses in this study were performed using JMP for Windows version 12.2.0 (SAS Institute Inc., Cary, NC, USA).

### Ethics approval and consent to participate

This study was conducted according to the guidelines of the Declaration of Helsinki and the Ethical Guidelines for Medical and Health Research Involving Human Subjects in Japan. The study has received approval from the ethics committees of the Okayama University Graduate School of Medicine, Dentistry, and Pharmaceutical Sciences (authorization number: Ken 1809-022), and the need to obtain written informed consent was waived due to retrospective nature of the study, where participants consent was implied by an opt-out approach.

## Results

### Patient characteristics and MTX dosage

**Figure 1:**
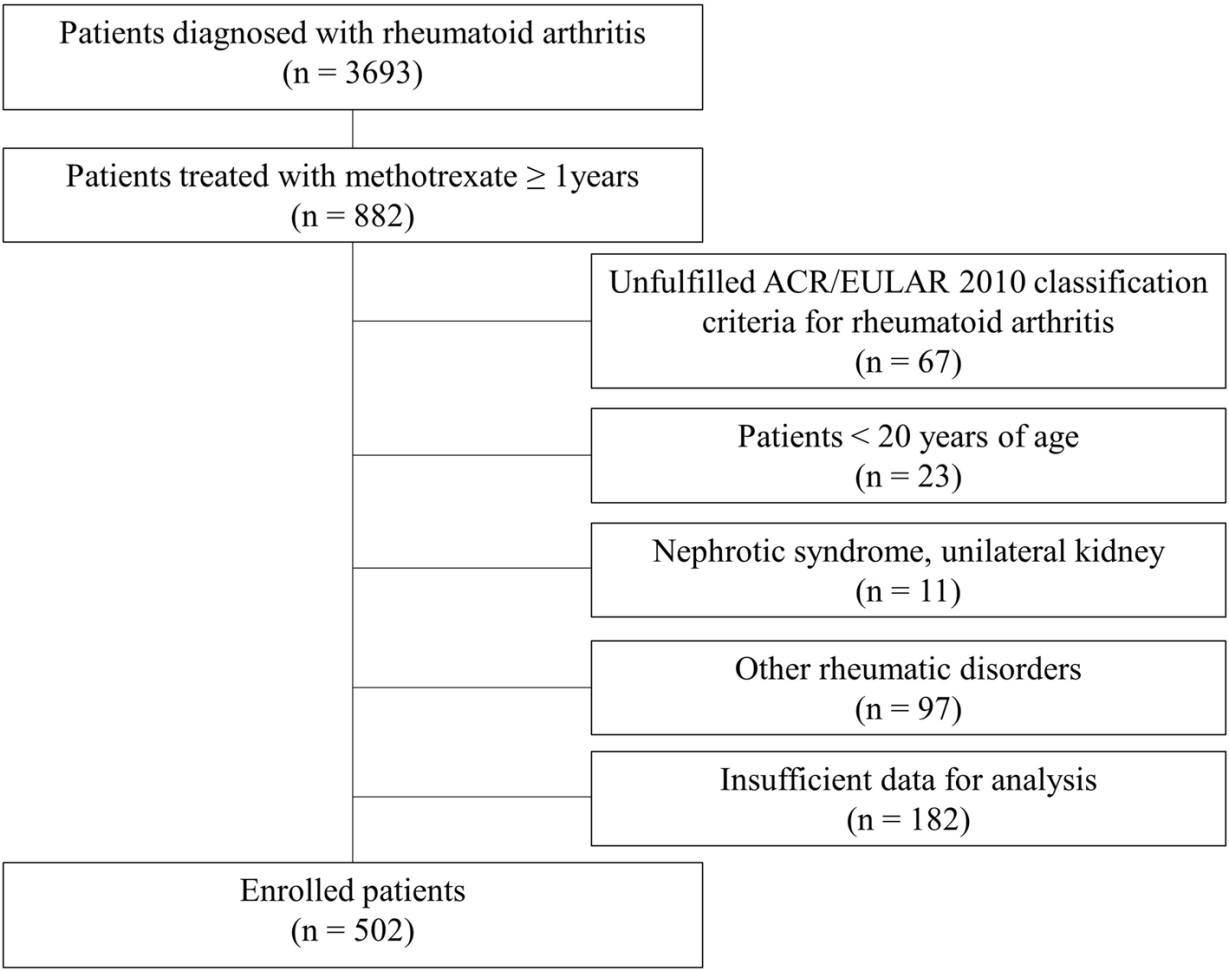
Patient selection flowchart.

The patient selection flowchart is shown in Fig. 1. The missing data (n = 182) derived from the following two reasons; (1) insufficient prescription data when patients did not receive prescriptions at our institution, but at other local clinics, (2) incomplete records during the transitional period of our electrical medical record system. The mean ± SD age of the enrolled 502 patients was 63 ± 13 years; 394 (78%) were female. The median (IQR) disease duration was 77 (31–159) months. The mean MTX dosage was 8.6 ± 3.0 mg/week, and bDMARDs or tsDMARDs were concomitantly used in 140 (27%) patients. The eGFR (mean ± SD) decreased by 1.2 ± 8.1 mL/min/1.73 m^2^ from 76.1 ± 17.3 mL/min/1.73 m^2^ during the 1-year observational period.

### Comparison among different MTX dosage groups

The enrolled patients were divided into three groups according to MTX dosage: < 8 mg/week, n = 186 (37%); 8–12 mg/week, n = 219 (43%); and ≥ 12 mg/week, n = 97 (19%).

A comparison of patient characteristics among these MTX dosage groups is shown in Table 1. Patients in the MTX ≥ 12 mg/week group were younger, had a higher baseline eGFR, and had a higher mean body weight than those in the MTX < 8 mg/week or 8–12 mg/week groups. The concomitant use of NSAIDs was significantly less frequent in patients in the MTX < 8 mg/week group than in those in the other two groups. The concomitant use of bDMARDs or tsDMARDs was more frequent in a dose-dependent manner with MTX but the difference between the groups was not significantly different. All disease activity scores were significantly higher with MTX use in a dose-dependent manner.

**Table 1:** Comparison of patients according to dosage: MTX < 8, 8–12, or ≥ 12 mg/week.

	All N = 502	< 8 mg n = 186	8–12 mg n = 219	≥ 12 mg n = 97	*p* value
Age, mean ± SD, years	63 ± 13	65 ± 13	61 ± 14	58 ± 13	0.0005*
Female, n (%)	394 (78%)	155 (83%)	172 (79%)	67 (69%)	0.02*
Disease duration, median (IQR), months	77 (31–159)	79 (24–196)	77 (33–134)	77(34–150)	0.03*
CKD, n (%)	86 (17%)	56 (30%)	27 (12%)	3 (3%)	< 0.0001*
Baseline eGFR, mean ± SD, mL/min/1.73 m^2^	76.1 ± 17.3	70.4 ± 17.9	77.3 ± 15.6	84.3 ± 16.1	< 0.0001*
ΔeGFR, mean ± SD, mL/min/1.73 m^2^	− 1.17 ± 8.1	− 0.19 ± 7.3	− 0.56 ± 8.6	− 4.48 ± 7.9	< 0.0001*
Body weight, mean ± SD, kg	54.4 ± 11.7	52.1 ± 9.7	55.1 ± 12.4	57.3 ± 12.7	0.0009*
BMI, mean ± SD, kg/m^2^	22.0 ± 3.7	21.6 ± 3.1	22.4 ± 3.9	22.2 ± 4.0	0.10
Sjögren’s syndrome, n (%)	24 (4.8%)	15 (8%)	7 (3%)	2 (2%)	0.03*
ILD, n (%)	21 (4%)	5 (3%)	11 (5%)	5 (5%)	0.44
Diabetes mellitus, n (%)	54 (11%)	22 (12%)	24 (11%)	8 (8%)	0.65
Hypertension, n (%)	180 (36%)	70 (39%)	78 (36%)	32 (33%)	0.74
Methotrexate dosage, mean ± SD, mg/kg	8.6 ± 3.0	5.6 ± 1.4	9.2 ± 1.1	13.1 ± 1.3	< 0.0001*
Folic acid, n (%)	337 (67%)	111 (60%)	154 (70%)	72 (74%)	0.02*
NSAIDs, n (%)	193 (38%)	59 (32%)	80 (37%)	54 (56%)	0.0003*
Prednisolone, n (%)	210 (42%)	66 (35%)	99 (45%)	45 (46%)	0.08
Prednisolone dose, mg ± SD	3.8 ± 1.2	3.5 ± 1.8	4.1 ± 2.8	3.9 ± 2.2	0.28
Salazosulfapyridine, n (%)	62 (12%)	28 (15%)	22 (10%)	12 (12%)	0.31
Iguratimod, n (%)	25 (5%)	5 (3%)	14 (6%)	6 (6%)	0.19
Bucillamine, n (%)	26 (5%)	8 (4%)	10 (5%)	8 (8%)	0.31
Tacrolimus, n (%)	73 (15%)	23 (12%)	30 (14%)	20 (21%)	0.16
Hydroxychlorquine, n(%)	0 (0%)	0 (0%)	0 (0%)	0 (0%)	–
b/tsDMARDs, n (%)	140 (27%)	47 25%	59 27%	34 (35%)	0.20
Infliximab, n (%)	34 (7%)	10 (5%)	15 (7%)	9 (9%)	0.46
Adalimumab, n (%)	17 (3%)	7 (4%)	6 (3%)	4 (4%)	0.77
Golimumab, n (%)	9 (2%)	0 (0%)	7 (3%)	2 (2%)	0.05
Certolizumab pegol, n (%)	8 (2%)	4 (2%)	1 (0.5%)	3 (3%)	0.17
Etanercept, n (%)	49 (10%)	20 (11%)	20 (9%)	9 (9%)	0.85
Tocilizumab, n (%)	9 (2%)	3 (2%)	4 (2%)	2 (2%)	0.96
Abatacept, n (%)	12 (2%)	3 (2%)	5 (2%)	4 (4%)	0.42
Tofacitinib, n (%)	4 (0.7%)	0 (0%)	1 (0.5%)	3 (3%)	0.016*
RASi, n (%)	80 (16%)	35, (19%)	33 (15%)	12 (12%)	0.33
DAS28-ESR, mean ± SD	3.1 ± 1.1	3.0 ± 1.0	3.1 ± 1.0	3.4 ± 1.2	0.04*
DAS28-CRP, mean ± SD	2.4 ± 0.9	2.2 ± 0.8	2.4 ± 0.9	2.7 ± 1.1	0.0019*
SDAI, median (IQR)	5.8 (2.8–9.3)	5.3 (2.2–8.6)	5.7 (3.4–9.3)	6.8 (3.2–11.6)	0.0032*
CDAI, median (IQR)	5.4 (2.5–8.6)	5.0 (2.0–8.1)	5.4 (3.1–8.6)	6.4 (2.7–10.7)	0.0040*
HAQ, median (IQR)	0.4 (0–0.7)	0.4 (0.1–0.8)	0.4 (0–0.6)	0.4 (0.1–0.8)	0.18
Proteinuria, n (%)	7 (1%)	3 (2%)	3 (1%)	1 (1%)	0.92
WBC, mean ± SD, /µL	6.25 ± 2.23	5.99 ± 2.37	6.35 ± 2.21	6.49 ± 1.97	0.13
Hemoglobin, mean ± SD, g/dL	12.7 ± 1.4	12.7 ± 1.4	12.6 ± 1.4	12.7 ± 1.4	0.92
Platelet count, mean ± SD, 10^3^/µL	249 ± 73	237 ± 70	255 ± 78	255 ± 64	0.03*
Blood urea nitrogen, mean ± SD, mg/dL	15.3 ± 4.4	16.0 ± 5.1	15.1 ± 4.0	14.6 ± 3.5	0.03*
Serum creatinine, mean ± SD, mg/dL	0.68 ± 0.16	0.72 ± 0.18	0.66 ± 0.14	0.64 ± 0.12	< 0.0001*
ESR, mean ± SD, mm/h	30 ± 24	31 ± 26	30 ± 24	31 ± 23	0.84
CRP, mean ± SD, mg/dL	0.66 ± 1.26	0.55 ± 1.16	0.68 ± 1.30	0.83 ± 135	0.20

*BMI* Body Mass Index, *b/tsDMARDs* biological/targeted synthetic disease-modifying antirheumatic drug, *CDAI* clinical disease activity index, *CKD* chronic kidney disease, *CRP* C-reactive protein, *DAS28* 28-joint Disease Activity Score, *eGFR* estimated glomerular filtration rate, *ESR* erythrocyte sedimentation rate, *HAQ* Health Assessment Questionnaire, *IQR* interquartile range, *NSAIDs* nonsteroidal anti-inflammatory drugs, *RASi* renin-angiotensin system inhibitor, *SD* standard deviation, *SDAI* Simplified Disease Activity Index, *WBC* white blood cells.

*p < 0.05 for comparison between patients with different MTX dosage groups.

### Change in eGFR among different MTX dosage groups

**Figure 2:**
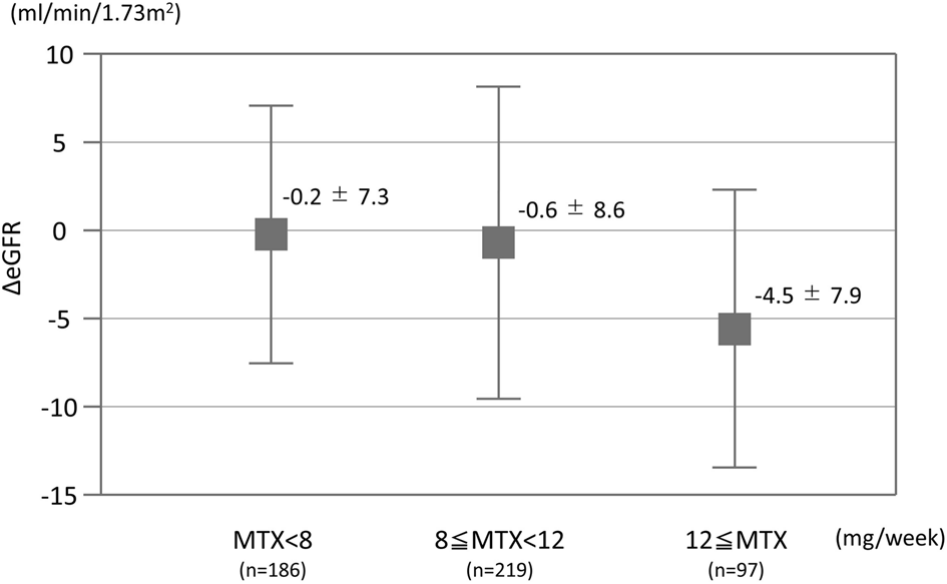
Change in eGFR over 1-year period among different MTX dosage groups.

The eGFR in patients treated with MTX < 8 mg/week, 8–12 mg/week, and ≥ 12 mg/week decreased by 0.2 ± 7.3, 0.6 ± 8.6, and 4.5 ± 7.9 mL/min/1.73 m^2^/year, respectively (*p* < 0.0001; Fig. 2). In the multivariate analysis, the following variables were selected as confounding factors based on the results of univariate analysis and previous reports: age, sex, disease duration, body weight, baseline eGFR, hypertension, diabetes, concomitant use of NSAIDs and bDMARDs/tsDMARDs, and DAS28-CRP^4,6–8^. After the adjustment for confounding factors using multiple linear regression analysis, the use of MTX < 8 mg/week did not significantly change the eGFR (beta-coefficient: − 0.86; 95% confidence interval [CI] − 0.65 to 2.36; p = 0.26) but that of MTX ≥ 12 mg/week was still correlated with a statistically significant decrease in eGFR (beta-coefficient: − 2.46; 95% CI − 4.30 to − 0.62; p = 0.0089) in contrast to MTX 8–12 mg/week use (Table 2). In addition, we observed the change of eGFR in the MTX ≥ 12 mg/week group patients for the 5-year extended period to ascertain the effect could accumulate. We could collect follow-up data of 62 patients out of the 97 patients who received MTX ≥ 12 mg/week. The eGFR in patients treated with MTX ≥ 12 mg/week decreased 13.8 ± 10.4 mL/min/1.73 m^2^ over 5-year period.

**Table 2:** Association of MTX dosage with change in eGFR assessed using multiple linear regression analysis.

	βcoefficient	SD	*p* value	95% confidence interval forβ
MTX < 8 mg/week (vs. 8 ≤ MTX < 12 mg/week)	0.86	0.77	0.26	− 0.65 to 2.36
MTX ≥ 12 mg/week (vs. 8 ≤ MTX < 12 mg/week)	− 2.46	0.94	0.0089	− 4.30 to − 0.62

Age, sex, disease duration, body weight, baseline eGFR, hypertension, diabetes, concomitant use of NSAIDs, concomitant use of b/tsDMARDs, and DAS28-CRP were used in the multiple linear regression models to adjust for confounding factors related to MTX dosage and renal impairment.

*b/tsDMARDs* biological/targeted synthetic disease-modifying antirheumatic drug, *CRP* C-reactive protein, *DAS28* 28-joint Disease Activity Score, *eGFR* estimated glomerular filtration rate, *NSAIDs* nonsteroidal anti-inflammatory drugs.

## Dicussion

In the present study, eGFR decreased by 1.2 mL/min/1.73 m^2^ over 1 year, but the larger decrease was seen in a higher dose MTX administration.

Renal impairment may persist over the course of treatment regardless of a disease duration in patients with RA receiving higher dose MTX. Significant nephrotoxicity occurs in 2–12% of patients treated with high-dose intravenous MTX for malignancy^14^. Nephrotoxicity is reportedly caused by crystal nephropathy due to the presence of MTX and its metabolites in the renal tubules; therefore, monitoring of plasma MTX concentrations, hydration, and urine alkalinization are recommended during treatment^14^. In cases of low-dose MTX treatment for RA, renal impairment caused by MTX is not mentioned in clinical practice guidelines^1,13,15^. However, there are some previous reports on renal impairment in RA patients upon the initiation of MTX^9,10,16^. Our study evaluated the change in eGFR during the previous 1 year in patients with a mean disease duration of 10 years. Therefore, renal impairment due to low-dose MTX can persist and progress over the long term after the initiation of MTX. MTX dosage was not reportedly associated with the detection of an abnormal eGFR (< 90 mL/min/1.73 m^2^)^10^. However, this previous report showed that eGFR in patients taking MTX > 15 mg/week (n = 29) and those taking MTX < 15 mg/week (n = 72) decreased by 6.8 and 8.8 mL/min/1.73 m^2^, respectively, during the mean 4-year observational period. The adequate sample size of our study enabled confirmation of the statistically significant differences in dose dependency even after the adjustment for confounding factors.

CKD was detected in 17% of the enrolled patients in this study. Previous reports showed that CKD occurred slightly more frequently in patients with RA than in the general population^5,17–20^. The prevalence of CKD in the present study was consistent with that in previous reports^5,19,20^. Because the administration of MTX should be avoided in RA patients with renal failure, a lower prevalence of CKD was expected in the present study than in these previous reports. Although risk factors for renal impairment such as some drugs and chronic inflammation have been reported^4,6,7,20^, MTX may also be an important risk factor for CKD in patients with RA. Although a decrease by 4.5 mL/min/1.73 m^2^/year seems small, the accumulated effects might be considerable and may lead to significant progression of CKD, which are demonstrated in our study by the 13.8 mL/min/1.73 m^2^ mean decrease of eGFR over 5 year period in patients with higher dose of MTX.

There are some limitations to this study. First, the MTX dosages were smaller than those in other clinical studies^21,22^. In Japan, the maximum recommended dose of MTX is 16 mg/week, which more than half of Japanese patients with RA cannot tolerate^13^. Therefore, our results suggest that patients treated with MTX should be monitored for renal impairment even in common clinical situations. Second, the mean MTX dosage was calculated during the observational period; thus, fluctuations in dosage were not considered. Although some patients may reduce the MTX dosage due to the progression of renal impairment, our result was not overestimated because these patients would be categorized into lower dosage groups.

In conclusion, careful monitoring of renal function should be required in treatment with MTX ≥ 12 mg/week among RA patients.

## Data Availability

The datasets used and analyzed during the current study are available from the corresponding author upon reasonable request.

## Abbreviations

RA: Rheumatoid arthritis
csDMARDs: Conventional synthetic disease-modifying antirheumatic drugs
MTX: Methotrexate
CKD: Chronic kidney disease
NSAIDs: Nonsteroidal anti-inflammatory drugs
eGFR: Estimated glomerular filtration rate
BMI: Body Mass Index
bDMARDs: Biological DMARDs
tsDMARDs: Targeted synthetic DMARDs
HbA1c: Hemoglobin A1c
WBC: White blood cell
Hb: Hemoglobin
s-Cr: Serum creatinine
ESR: Erythrocyte sedimentation rate
CRP: C-reactive protein
DAS28: 28-Joint Disease Activity Score
SDAI: Simplified Disease Activity Index
CDAI: Clinical Disease Activity Index
HAQ: Health Assessment Questionnaire
SD: Standard deviation
IQR: Interquartile range
CI: Confidence interval
